# Gleanings from Our Exchanges

**Published:** 1872-04-01

**Authors:** 


### Gleanings from Our Exchanges.

On the Treatment of Hemorrhoids in Pregnant and Puerperal Women. — Dr. Fordyce Barker contributes an article on this subject to the *American Practitioner* for March. Contrary to the general teaching he always gives aloes in hemorrhoids of pregnant women, whether accompanied by constipation or diarrhœa, arguing that the aloes exercises a tonic influence on the muscular coats of the vessels of the rectum and colon. During the last two weeks of gestation he always combines the aloes with the extract of belladonna. The following is a frequent prescription:

℞ Pulv. Aloes Soc.,

Sapo. Cast., aa., ℈ji.

Ext. Hyosciami, ʒ ss.

Pulv. Ipecacuan, gr. v.

M. Fit Pil. (argent) No. 20. S. on morning and evening.

When a patient is anæmic he adds one scruple of sulphate of iron to this prescription.

When the patient has an irritable rectum, with small teasing, thin evacuations, he substitutes for the hyosciamus a small quantity of the aqueous extract of opium and a smaller quantity of the aloes.

When he is called to a case during labor, and the tumors come down and are troublesome, he tries the method of Dewces, returning the tumors and pressing over the anus with a napkin during the expulsion of the head. This method however, he says, frequently fails in keeping the tumors back. In that case he tries the method of Van Buren, anesthetizing the patient and with the thumbs dilate the sphincter as wide as possible. He then directs the following:

℞ Ung. Gallæ co.,

Ext. Opii. aq.,

Sol. Ferri persulph.,

M. Ft. Ung.

If the woman's bowels do not move, he does not give castor oil, claiming that it is the worst laxative possible when there is a tendency to piles. He gives the following:

℞ Magnesia Sulph.,

Magnesia Carb.,

Potas. Sup. Tart.,

Sulphur, Sublim., aa. ℥ ss.

Mix thoroughly. S. one, two, or three teaspoonfuls before eating in the morning.

Chronic Otorrhœa.—Dr. A. W. Calhoun describes the treatment of Dr. Weber, of Berlin, in this disease, says that he seldom directs the syringing of the ear with tepid water, as the few drops of water remaining often produce an acute catarrh, thus complicating the disease. He dries the ear with a fine camel-hair pencil and then fills the external meatus with pure alcohol, first, tipping the head slightly to one side and rubbing and gently pressing upon the Tragus. After about five minutes the alcohol is turned out and the meatus is filled with charpie. Continue this treatment three times a day until the discharge has markedly diminished, afterwards twice a day until the disease has disappeared. For a while after its disappearance it is well to continue the medicine once a day. The success of this treatment he claims to be very great.—*Georgia Med. Companion.*

Prolonged Expiration.—Dr. Armor contributes an article on this subject to the New York *Medical Journal*, referring to the physiology of the lung, he calls attention to the fact that expiration is entirely a passive movement, and is accomplished by the recoil of the elastic fibers which enter into the composition of the lung tissues. Anything which weakens these fibers will diminish the expiratory forces and lengthen the time of expiration, as is well seen in emphysema. In the *pretubercative stage* of consumption the nutrition of the lung is imperfect, and these fibers are weakened, hence the prolonged expiration. Prolonged expiration is then by no means diagnostic of tubercle or even of incipient phthisis; general anæmia from any cause may produce it, but taken in connection with the rational symptoms generally present indicating a defective nutrition, it is a sign of very great value as indication of a *tendency to tubercular* deposit.

A new Speculum is figured in the *Lancet* for February. The speculum opens like a bivalve speculum, each valve being made of stout German silver wire bent like a letter M. When the speculum is to be introduced a rubber band is slipped over it which confines the four parts in a narrow compass; after the introduction the band is slipped off when the four points separate by their own resiliancy, exposing the parts, the inventor claims, the most satisfactorily of any speculum which has yet been shown.

Digestive Power of Pepsin.—A contributor to the *Am. Journ. of Pharmacy* describes his method of preparing pepsin, and says that one half grain of the pepsin added to two ounces of water acidulated with hydrochloric acid dissolved 250 grains of coagulated albumen, to the solution was added another ounce of acidulated water and 250 grains more of albumen was dissolved. He continued adding acidulated water and albumen until he had dissolved 1500 grains of albumen, and not even then was the solvent power exhausted.

Beef Extract.—The iconoclast is among our idols. Chemistry proved to us some time ago, that gelatine in all its forms was not nutritious. The English doctors are rebelling against alcoholics; and here comes along an irreverant gentleman, Prof. Muller, who claims not only that beef extract has no nourishment whatever, but that the potash salts contained in the essence will, if taken into the system in large quantities, diminish the capacity of the red blood corpuscles for carrying oxygen and so have a positive debilitating effect.

It is said that a mixture of hydrochloric acid and pepsin will, contrary to the general opinion, dissolve calomel.

### NECROLOGIST'S REPORT.

(*Continued from page* 96.)

Dr. Otho Bonser — *Class of* 1868. — Dr. Bonser was the son of Jesse and Agnes Bonser, and born in Clermont County, Ohio, on the banks of the Ohio River, August 6th, 1844. When he was but three years old, his parents removed to Lee County, where a year later his father died, leaving Otho, with three sisters, one older, the other two younger than he, to the care of Mrs. Bonser, who, through the rascality of Mr. Bonser's debtors, was soon made penniless. Mrs. B., however, managed to keep her children together and send them to school until Otho was ten years old, when a gentleman in the neighborhood who had taken a liking to the studious boy, induced his mother to let Otho come into his family, promising to give him a finished education. He remained with this gentleman, however, but two years, when he left him and hired out to a man living near Mrs. B., in order to be nearer his mother; "for he was always a great mother's boy," writes his mother.

He still attended school in the winters, working in the summers, until he was sixteen years old, when he engaged to teach a three months school.

We learn that at this time his mother was a second time married, and they were all living in Iowa, though at what time these occurrences came about, we are not informed.

Before his first school was out the rebellion had broken out; and as soon as his school term closed, and before he was seventeen years of age, he enlisted. July 15th, 1865, he entered the service in Company K, 7th Iowa infantry. Just after the battle of Belmont he was sent home with typhoid fever. In three months he was able to rejoin his regiment on the eve of a heavy engagement, immediately after which he was promoted to Orderly Sergeant, and came home at the end of his three years' enlistment as Second Lieutenant. At the expiration of the thirty days' furlough allowed veterans, he returned to the field as a First Lieutenant, where he remained until the close of the war, arriving home in July, 1865.

His mother writes us as follows: "He was a great favorite with all the boys; if any got into trouble, he was the one to settle it. He bought and read a great many good books while there. He carried his Bible with him, and visited the sick and wounded whenever opportunity permitted. He gave his Bible on one occasion to a sick soldier. On another, to a wounded one he gave almost all the money he had. He was converted when ten years old, and united with the Congregational Church; and until his death he lived a consistent Christian."

In September, 1865, he began the study of Medicine at the University of Michigan, entering as a student of the Faculty. Writes a chum of his at Ann Arbor: "It was there the writer first met him, and there the friendship was formed which grew deeper and stronger until arrested by the Doctor's death. Our college life there was what it is with all or almost all western medical students. We were both poor and hard run; but one never wanted for a shilling if the other possessed so much. Bonser was a hard, conscientious student; he never slouched over his work. One incident of his life there it may be well to mention, as exhibiting something of his character. He was engaged to be married to a young lady at that time going to school in Ohio. About the holidays he received a letter from her informing him that she had been mistaken in her feelings towards him, and that while she loved no other, yet she could not conscientiously marry him. To a man of his deep nature, this was the heaviest blow he could receive. The future looked black enough. Poor, and with no influential or able friends, too delicate for hard labor, his profession to get, and even the love of woman denied him; many men would have gone entirely to the bad. For twenty-four hours his suffering was terrible. 'Heaven alone knows,' said he, 'how I love that woman!' Then to work; no further outcry; no change, except that he worked the harder."

The summer of 1866 he spent on a farm in Iowa, near his mother and sister. He attended the session of 1866-7 at the Chicago Medical College. The ensuing summer he was with Prof. R. J. Patterson at Batavia, Ill., in the Bellevue Place Insane Asylum. The winter of 1867-8 again found him at the Chicago Medical College and in Mercy Hospital.

In the spring of 1868, shortly after his graduation, he went to Kansas. Settling penniless and in debt at Aubrey, Johnson Co., he soon gathered friends and patrons about him, and in six months had perhaps as good a country practice as any man in his county.

"While there," writes Dr. S. T. Odde, a former classmate, "it was my good fortune, practicing medicine in a little village ten miles distant, to often meet him in consultation, and always with benefit both to the patient and myself. In February, 1869, I received a note from him: 'O, I am "under the weather" a little; have been so for two weeks, and do n't get any better; wish you 'd come up and "straighten me out."' Obeying the call at once, I found he had taken a severe cold in riding through a storm to an obstetric case a few nights previous, and a bad cough was the result. His father had died of Tuberculosis, and we feared a like fate for him. A few days showed our suspicions but too well founded, and he gradually sank until April 10th, 1869, when he quietly breathed his last (he was thus 24 years, 8 months and 4 days old). He was nature's nobleman — a brave, patient, intelligent, hard worker in the cause of God and humanity. We know he has his reward."

While in the class of '68 of the Chicago Medical College Bonser was, I think, respected by every one. He was modest in his disposition, and not bold to make acquaintances on short notice, and with many of the class he was not very thoroughly acquainted. But those who knew him best respected him most, and loved him; and he left college with no feeling but of kindness in anyone towards him.

Prof. Patterson says, in a letter to the Necrologist: "Bonser was a noble fellow; kind, generous, industrious, a great soul, a true man, a true physician, a devoted Christian." Of whom could more be said?

Dr. B. was, at the time of his death, engaged to be married to a very intelligent lady in Aubrey.

